# Telepresence robots as facilitators of physical exercise during COVID-19: a feasibility and acceptance study

**DOI:** 10.3389/fpubh.2023.1277479

**Published:** 2023-12-27

**Authors:** Abdullah Addas

**Affiliations:** ^1^Department of Civil Engineering, College of Engineering, Prince Sattam Bin Abdulaziz University, Alkharj, Saudi Arabia; ^2^Landscape Architecture Department, Faculty of Architecture and Planning, King Abdulaziz University, Jeddah, Saudi Arabia

**Keywords:** COVID-19, physical exercise, telepresence robots, mental health, public health

## Abstract

The COVID-19 pandemic and associated restrictions on mobility and access to green space have disrupted exercise habits worldwide. According to the World Health Organization (WHO), approximately 1.4 billion adults were insufficiently physically active in 2016, with detrimental impacts on health. The proposed study investigated the use of telepresence-robot-based personal trainers to facilitate remote exercise during the pandemic-related lockdowns. Several adults aged 18–65 were recruited for a four-week intervention and thorough research investigation. The intervention involved one-hour outdoor exercise sessions held three times per week in a local park with a human instructor connected via a telepresence robot. Surveys assessed perceptions of social presence, usability, the intention to use the robot and the psychological benefits of access to green space. System logs tracked participation and technical errors. At baseline, 30% of the participants met the WHO physical activity (PA) recommendations, compared to 80% after the intervention. The study shows significant increases in many parameters. These are perceived in social presence (*p* < 0.021), usability (*p* < 0.04), intentions for long-term use (*p* < 0.05), and the mental health benefits of accessing green spaces (*p* < 0.013). Attendance was found to be 90%, with a 7% technical failure rate. This investigation demonstrates the promise of telepresence robots for safely providing remote access to green spaces. They can be used to facilitate exercise during public health crises, overcoming the barriers to maintaining PA.

## 1. Introduction

### 1.1. Background

**Figure 1:**
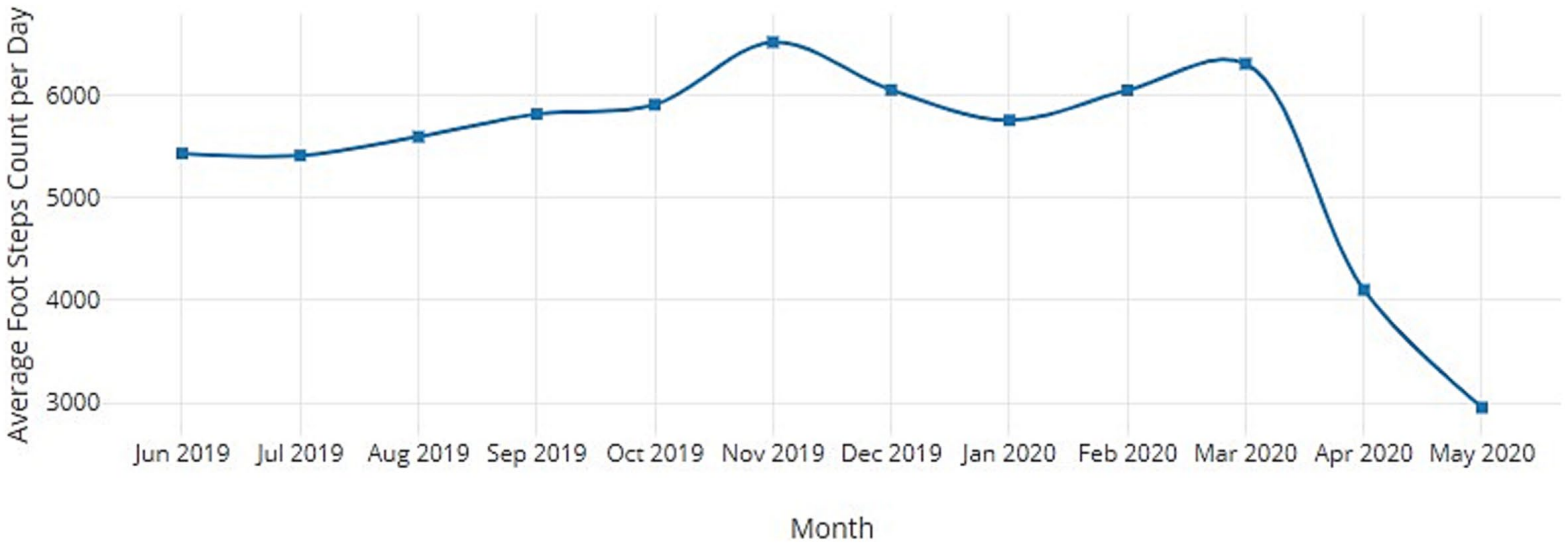
Physical activity of an average human for pre and post COVID-19.

The COVID-19 pandemic profoundly impacted populations worldwide, necessitating restrictive public health measures to curtail viral transmission, which disrupted daily living. Mobility restrictions, social distancing, lockdowns, and the closure of parks and recreation facilities have presented formidable barriers to maintaining physical activity (PA) participation during this crisis (1). Physical inactivity was already a global issue before COVID-19, with the World Health Organization (WHO) estimating in 2016 that 1.4 billion adults failed to meet the recommended PA levels, making it one of the leading risk factors for mortality and fueling the rising prevalence of noncommunicable diseases (2). The COVID-19 pandemic has only exacerbated this problem, with early studies showing dramatic declines in PA during the initial lockdowns, including a 48% reduction in sports participation and a 58% decrease in outdoor exercise compared to pre-pandemic levels (3). Smartphone step count data from the past 12 months indicate that the average daily steps were consistent between June 2019 and May 2020. However, there was a noticeable drop of approximately 2,500 steps per day during April and May 2020, as illustrated in Figure 1.

These trends are concerning given the well-established physical and mental health benefits of regular PA. Physical inactivity is associated with a higher risk of cardiovascular disease, diabetes, cancer, and all-cause mortality, accounting for an estimated 5 million deaths worldwide each year (4). Exercise confers protection against chronic illnesses by impacting weight management, blood pressure, insulin sensitivity, lipid profiles, immune function, and inflammation (5). Additionally, PA has demonstrated antidepressant and anxiolytic effects, which can mitigate the adverse psychological effects of isolation, grief, stress, and sedentary behavior during prolonged lockdowns (6). However, realizing these multifaceted health benefits hinges on having the motivation, resources, and access to maintain routine exercise, which COVID-19 constraints have severely limited.

While the resumption of standard PA patterns is anticipated post-pandemic, the crisis highlights the need for technologies that facilitate remote exercise, as well as access to recreation spaces, when mobility is disrupted. Virtual fitness solutions have burgeoned but require more key social dimensions and environmental engagement. In contrast, telepresence robots, which enable remote embodied interactions through a movable videoconferencing display, offer a novel approach to overcoming barriers to active lifestyles during public health emergencies. Telepresence robots have been effectively utilized in healthcare applications (7), but their use to promote PA and access to nature still needs to be explored.

Pilot initiatives before the pandemic provided initial evidence that telepresence robots could enable immersive park experiences. For example, Double Robotics and California State Parks successfully trialed remote park exploration via telepresence robots, allowing people to hike trails and interact with rangers Double Robotics (8). Such an environmental telepresence could mitigate some of the psychological harms of isolation from green spaces, which have well-documented restorative benefits for mental health and cognition (9). Additionally, previous research found that robots can enhance exercise motivation and performance. When paired with a virtual avatar coach, people spent more time running and reported greater enjoyment (10). Telepresence robots may have similar motivational effects while increasing perceived social presence and engagement.

The main contributions of the proposed investigation are as follows:

- Emerging evidence warrants further inquiry into the feasibility of using telepresence robots to facilitate remote PA within natural environments from which people are excluded during public health crises;
- We quantitively evaluate this novel application’s acceptance, experience, and effectiveness, informing design optimizations for long-term viability;
- Elucidating how the environmental and social telepresence afforded by an embodied robotic platform can enhance adherence, autonomy, and psychological responses to exercise could guide technological development and policy related to remote active lifestyles;
- The proposed study was devised to provide an initial systematic investigation of telepresence robots as tools with which to foster increased PA amidst the unprecedented constraints of the COVID-19 pandemic and the protracted periods of physical inactivity it caused.

### 1.2. Literature review

The COVID-19 pandemic has created an urgent need to find solutions that can support the maintenance of regular physical activity amidst constraints on mobility and access to exercise facilities. A growing body of literature across diverse fields, including human-robot interactions, telehealth, exercise science, the psychology of motivation, and environmental health, has investigated the applications of telepresence robotics that allow for remote embodied interactions as potential tools to overcome the barriers to active lifestyles imposed by public health crises. This review synthesizes the key findings on user acceptance and experiences with telepresence robots in healthcare and education, embodiment factors influencing human-robot exercise engagement, the effects of virtual coaches and social facilitation on motivation, and the benefits of simulated natural environments for wellbeing.

Several studies have identified essential considerations around usability, navigation, and control interfaces that broadly influence the acceptance and adoption of telepresence robots (11–14), which can inform device optimization in exercise contexts. Smooth maneuverability and camera operations impacted the perceived ease of use and the intention to employ healthcare telepresence robots (15). Difficulties with low-level control reduced the acceptance of a hospital delivery robot by staff (16). On the other hand, a homecare robot was perceived as valuable and satisfactory when the controls enabled safe and effective navigation (17). These findings highlight the need to refine exercise telepresence robots’ movement and camera controls to ensure seamless remote mobility.

Additionally, trust-mediated acceptance and willingness to rely on healthcare robots emphasize the importance of careful technical management to maximize uptime and minimize failures (18). The perceived sociability of the robot’s appearance and etiquette capabilities also led to better user evaluations (19, 20). Implementing humanlike cues and socially appropriate communication aligned with exercise norms could improve engagement. Several studies have evaluated telepresence robots’ abilities to enhance social connection and communication in medical and academic settings, demonstrating their viability for improving access and psychological outcomes in remote service delivery. In healthcare contexts, social telepresence robots mitigated feelings of isolation for hospitalized children (21), increased interactions between infants and parents (22), enabled nursing students to interact with patients (23), and helped to remotely assess cognitive function in older adults during the pandemic (24). Patients, families, and staff overwhelmingly reported that the robots were easy to use and improved access, socialization, mood, and care quality (25–27). In education, remote learners felt a more significant social presence, rapport, enjoyment, and learning with instructors when telepresence robots were used instead of videoconferencing (28–30). Telepresence facilitated more natural gazes, mobility, and nonverbal cues, overcoming the limitations in relational connection encountered when using standard video. These applications demonstrate that telepresence robotics enable more embodied, socially engaging, and responsive interactions, which are crucial for delivering personalized services remotely. Exercise training could similarly benefit from heightened instructor presence and environmental access.

The sense of human connection and physical embodiment, which is uniquely provided by telepresence robots in the field of telecommunication, could enhance exercise performance, experience, and motivation. A virtual agent presented through a robotic interface increased treadmill running time and self-reported motivation compared to audio guidance alone, and was associated with heightened perceived agency and relational bonds attributed to physical embodiment (31). Incorporating an instructive avatar within the telepresence robot may augment adherence and engagement. Additionally, a robotic embodiment can lead to increased exercise intensity compared to technological mediation alone. Participants cycled faster when pacing against a physically present robot than when chasing a virtual avatar, indicating greater motivation from tangibility (32). In competitive tasks, the mere presence of a robot observer increases effort and arousal as compared to virtual observation (33). Virtual coaches also improved running performance and form but embodied robots enabled more natural social facilitation (10, 34, 35). The motivational and social facilitation effects of a robotic presence could be leveraged to promote active lifestyles.

Interacting with nature via telepresence technology could further augment the well-demonstrated restorative properties of outdoor exercise. Access to green spaces provides cognitive, emotional, and physiological health benefits, including stress reduction, mood enhancement, and improved immune function and life satisfaction (36–45). Virtual simulations of natural sights and sounds can confer similar advantages when the opportunities for actual experiences in nature are restricted (46–48). Telepresence robotics may optimize these outcomes by enabling multisensory immersion within real physical environments, overcoming the limitations encountered with virtual reality. In an early demonstration, PARKbot allowed remote park visitors to control their exploration and interact with sights, sounds, and staff through its camera, speakers, and iPad interface (49). Users reported deriving positive emotions and high satisfaction from the vicarious nature experience and social interactions afforded by the telepresence platform. Telepresence exercises in parks and green spaces could provide enriched psychological benefits, surpassing those of virtual simulations.

Despite these promising indications, quantitative data that directly highlight the impact of telepresence robotics on exercise adherence, performance, experience, and acceptance compared to other modalities under pandemic constraints remain scarce. While locomotion interfaces such as treadmills offer more physical engagement than seated telepresence (50), they lack environmental verisimilitude. Initial surveys found moderate willingness to use home treadmill systems paired with nature videos during lockdowns (51). Further comparison studies are needed to examine diverse exercise modalities under different mobility restrictions. Additionally, the existing work has focused on single-session studies rather than long-term interventions (32, 39). Field evaluations of sustained engagement and outcomes are critical next steps for clarifying the viability of telepresence robots for supporting remote active lifestyles during viral pandemics. The results from these regular exercises include the measurement of enjoyment, ease of use, and other metrics. The idea of adding telerobotics is the key intervention of the proposed research work. A similar study was carried out by (52), where long-term care was included to minimize the loneliness associated with COVID-19. (53), discuss physical presence and participants’ enjoyment of the sense of physical activity with easy-to-use mobile devices and training exercises.

In summary, the existing evidence indicates that telepresence robotics could provide an engaging embodied medium for delivering remote physical activity interventions, with potential advantages over other virtual methods (54–56). However, further research is needed to quantify the impact of telepresence exercise on adherence, performance, psychological responses, and technological acceptance in the context of the mobility limitations imposed during public health crises. Optimizing this emerging application based on systematic data can help sustain active lifestyles and the associated health benefits when access to facilities, instructors, and natural environments is precluded, but the maintenance of an active lifestyle remains vital.

### 1.3. Study rationale and objectives

The COVID-19 pandemic constituted an unprecedented global public health crisis, necessitating restrictive measures to control viral transmission with severely limited mobility and access to exercise facilities. Physical inactivity has markedly increased during the pandemic, exacerbating the already high worldwide rates of noncompliance with physical activity recommendations (2). This dramatic reduction in active lifestyles has significant implications for population-level health across all age groups. Regular physical activity confers well-established physical and mental health protective benefits. In contrast, physical inactivity is linked to higher risks of cardiovascular disease, diabetes, cancer, depression, cognitive decline, and premature mortality (4). The COVID-19 pandemic threatens to undo the public health gains in increasing exercise rates that were achieved before the crisis.

With the protracted nature of the pandemic and the periodic implementation of social distancing measures expected to continue for the foreseeable future, innovative solutions are urgently required to find ways for people to meet physical activity safety needs when access to gyms, fields, parks, and recreation centers is restricted. Some initial evidence indicates that telepresence robotics may offer a promising approach for facilitating remote active lifestyles during public health emergencies by enabling access to instructors and natural environments otherwise inaccessible due to mobility restrictions. However, improvement is required in terms of research that systematically evaluates the feasibility, acceptance, experience, and effectiveness of telepresence exercise interventions.

Quantitatively investigating this emerging application is critical to clarifying its viability and potential advantages compared to other virtual methods for supporting remote physical activity. The overarching goal of this study was to conduct an in-depth mixed-methods evaluation of using telepresence robots to deliver structured exercise training during pandemic conditions that render traditional in-person options unfeasible. The main objectives of the proposed investigation are as follows:

- To assess the feasibility and technical performance of a telepresence robot exercise program, including participation rates, safety, and technological reliability;
- To measure user acceptance and experience, including perceived usefulness, ease of use, presence, engagement, enjoyment, and intent to continue the telepresence exercise;
- To evaluate physical activity outcomes, including the achievement of exercise recommendations and fitness indicators before and after the telepresence intervention.

The study sought to generate rich quantitative and qualitative data on utilizing telepresence robots to promote remote physical activity that is grounded in a real-world implementation. The findings can guide effective practices, technological optimization, and policies for leveraging telepresence robotics to foster resilient, active lifestyles and mental health during viral pandemics. Broader applications for improving equitable access to exercise and recreation through embodied telepresence are also highlighted.

## 2. Materials and methods

The study utilized a mixed-methods design incorporating surveys, interviews, system logs, and fitness assessments. The participants were adults aged 18–65 and were recruited through convenience sampling. The telepresence robot platform was the Double 3 model. The eight-week intervention involved thrice-weekly one-hour outdoor exercise classes in local parks led by a trainer via the telepresence robot. The quantitative data collected included system performance metrics, questionnaires on acceptance, and pre-post fitness tests; the qualitative data comprised interview feedback on user experience. The analysis included descriptive and inferential statistics, content analysis, and triangulation to comprehensively evaluate the telepresence exercise.

### 2.1. Study design

The study utilized a mixed-methods pre-post single-group design to evaluate the use of a telepresence robot for delivering a remote outdoor group exercise program. Mixed methods combining qualitative and quantitative data were used to comprehensively assess feasibility, acceptance, experience, and outcomes. The pre-post design enabled us to compare key metrics before and after the 8-week telepresence exercise intervention.

#### 2.1.1. Participants and setting

A convenience sample of 40 healthy adults aged 18–65 was recruited via email lists and social media from a large urban university community. The inclusion criteria were English fluency, no mobility impairments, and no regular structured exercise exceeding 150 min/week. The institutional review board of the university approved the study, and written informed consent was obtained.

The setting was neighborhood public parks within one mile of campus, providing an open outdoor exercise space. A different park was used each week to provide variety. The parks were selected based on the presence of suitable paths and sufficient cell signal strength to support the connectivity of the telepresence robot.

#### 2.1.2. Study protocol

Enrolled participants completed baseline assessments and then participated in a structured 8-week outdoor exercise program delivered remotely 3 times/week, with each session lasting 1 h, *via* the telepresence robot. The robot was driven on park paths by a certified fitness trainer who led cardiovascular, strength, and flexibility training tailored to individual fitness levels using bodyweight and portable equipment. Participants joined the class from home on their own devices via secure video software, exercising alongside the trainer and other participants who were live-streamed through the telepresence robot. Post-intervention assessments were conducted in Week 9.

### 2.2. Participants

A total of 40 healthy adults (20 female, 20 male) were enrolled in the study, with 28 (14 female, 14 male) completing the 8-week intervention and all assessments. Table 1 shows the participants’ demographic characteristics. The mean age was 41.5 years (SD: 12.4; range: 22–65). Most were white (73%) and in full-time employment (80%). Most (57%) had a bachelor’s degree or higher. All participants reported having no mobility limitations or health conditions that restricted exercise. The average self-reported exercise at baseline was 89.5 min/week (SD: 66.8; range: 0–240).

**Table 1:** Participant demographics.

Variable	Statistics
Age (years)	Mean 41.5 (SD 12.4) Range 22–65
Gender	Female 20 (50%) Male 20 (50%)
Ethnicity	White 21 Asian 14 Black 3 Other 2
Education	High school diploma 3 Some college 18 Bachelor’s degree 9 Graduate degree 10
Employment	Full-time 23 Part-time 12 Unemployed 5
Exercise (min/wk)	Mean 89.5 (SD 66.8) Range 0–240

Participants were recruited through posted fliers, social media, and email advertisements circulated among the university community, describing the study as assessing the use of an “interactive mobile robot” for exercise training. The inclusion criteria stated that participants had to be adults who engaged in low to moderate levels of activity with no mobility impairments or medical conditions that limited exercise. The interested respondents completed an online screening process covering demographics, exercise habits, health status, and comfort using mobile apps and videoconferencing. The eligible candidates were scheduled for an orientation where they provided written informed consent and completed baseline assessments before enrolling.

### 2.3. Telepresence robot platform

The Double Robotics’ Double 3 telepresence robot was selected as the mobile robotic platform to deliver the exercise intervention remotely. This model was chosen based on its balance of cost, durability, maneuverability, camera quality, and conversational features to suit the needs of a group outdoor exercise program.

**Figure 2:**
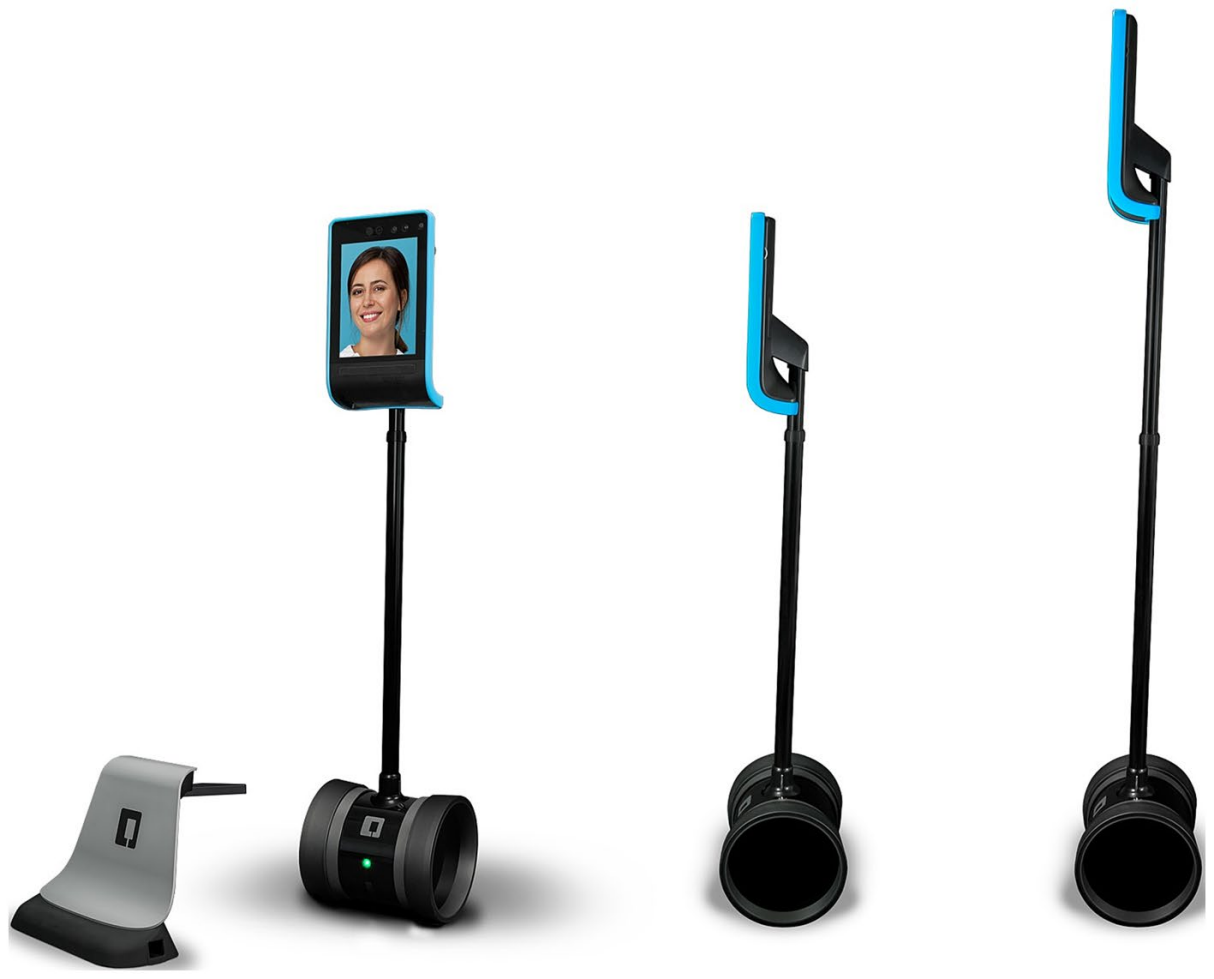
Double 3 telepresence robot with full kit.

The Double 3 has a lightweight aluminum frame on two wheels with an attached iPad to enable videoconferencing via 4G LTE. The iPad provides a 1080 p high definition (HD) camera livestream through a wide 120° field of view camera. Audio is captured through a 4-microphone array for high-fidelity sound transmission. Speakers allow the remote user to deliver clear audio. The iPad screen can be tilted up and down using a remote control to adapt the viewing angle. The Double 3 weighs 15 lbs., has a maximum speed of 3 mph, and has a battery life of 8 h per charge. Figure 2 shows the Double 3 telepresence robot.

The robot is controlled via an iOS or Android app connected over cellular data networks. Low-latency control allows for smooth driving and navigation. The app interface provides intuitive controls modeled on video game controllers. Buttons control forward, backward, left, and right driving. The camera can pan and tilt through a 330° range with sightline adjustable up to 6 feet high. The microphone and speaker volume are also adjustable.

The robot is turned on at the class location, automatically connecting with the iPad controller app to initiate a session. The instructor then drives the robot through the park using simple tap and swipe controls. Participants join remotely from their devices using the videoconferencing platform to observe the instructor and surroundings from the robot’s first-person camera perspective. Table 2 summarizes the Double 3’s specifications for delivering the telepresence exercise program.

**Table 2:** Double 3 telepresence robot specifications.

Feature	Specification
Dimensions	20″ tall x 15″ wide × 11″ deep
Weight	15 lbs
Speed	Up to 3 mph
Battery life	Up to 8 h continuous
Camera	1080 p with 120° field of view
Microphones	4-microphone array
Speakers	Dual integrated speakers
Connectivity	4G LTE cellular data
Control interface	iOS/Android app with tactile controls
Communication	Integrated videoconferencing over WiFi

The Double 3 provides key capabilities to enable remote group exercise, including two-way audiovisual communication, intuitive controls for driving over park terrain, adjustable viewing angles, and cellular connectivity to access outdoor locations. These features support an engaging, socially interactive exercise experience that mirrors an in-person class as closely as possible.

### 2.4. Study protocol

The 8-week telepresence exercise intervention consisted of three 60-min outdoor group workout sessions per week led remotely by a certified personal trainer via the Double 3 robot. The sessions took place in the late afternoon in neighborhood public parks within one mile of campus, and the location was varied each week to provide a diversity of scenery. The parks were selected based on the presence of suitable flat paths to support robot navigation and adequate cell service coverage to maintain connectivity.

At the start of each session, the trainer drove the robot to the designated park location and positioned it in an open area. The trainer followed a planned workout routine while navigating the robot throughout the park to showcase the surroundings. Participants joined the class virtually from their devices using the Zoom videoconferencing platform. Zoom provided secure multi-user video streaming and was accessible through a web browser or mobile app.

**Figure 3:**
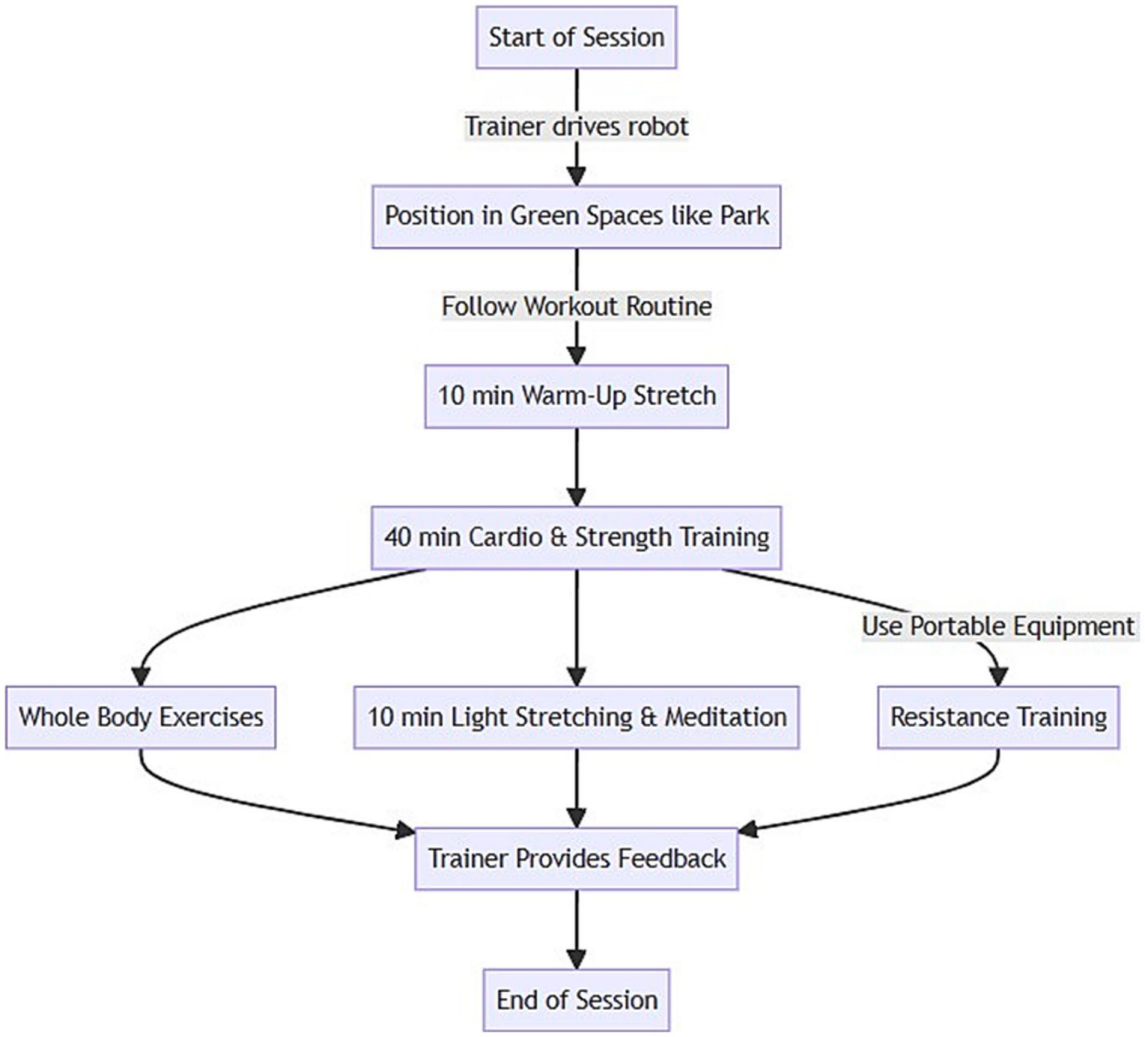
Flowchart of telepresence robot based exercise in green spaces by remote physical trainer.

The 60-min sessions consisted of a 10-min warm-up stretch, 40 min of cardiovascular and functional strength training, and a 10-min cool-down stretch. The trainer tailored the intensity levels and exercise modifications for everyone based on their fitness assessments. The cardiovascular training comprised interval walking, jogging, or running, following the robot along park paths. The resistance training used portable equipment such as resistance bands, lightweight dumbbells, and stability balls. The whole-body exercises targeted the major muscle groups. The final cooldown included light stretching and meditation. The trainer provided technique instruction, feedback, and encouragement modeled after in-person training throughout the class. Participants could converse with the trainer and their peers throughout the workout via videoconferencing. The whole flow of the process of this telepresence-robot-based exercise is illustrated in Figure 3.

All sessions were scheduled at the same time weekly for consistency. Before each session, participants received a Zoom link and location details. Attendance was recorded based on logins. If technical issues occurred, the trainer attempted to resume the session or schedule a make-up session. For safety, participants were advised not to exercise outdoors if the weather conditions were hazardous. The cell coverage in outdoor locations was verified in advance, but any connectivity losses during a session were documented. Participants were given a charging pack to maintain adequate smartphone battery life for the full hour. All the procedures and activities were approved by the university IRB and aligned with guidelines for outdoor exercise during the COVID-19 pandemic.

### 2.5. Quantitative and qualitative measures

Quantitative and qualitative measures were used to evaluate the feasibility, acceptance, user experience, technological performance, and physical activity outcomes associated with the telepresence exercise intervention.

The feasibility and technology metrics included:

- Attendance rates: percentage of total exercise sessions attended derived from videoconference login records;
- Attrition rate: percentage of participants lost to follow-up or dropping out;
- Safety incidents: number of falls, injuries, or other adverse events reported during sessions;
- Technical difficulties: system logs and trainer notes documented connectivity losses, battery failures, and app crashes.

Acceptability and experience were assessed according to the following metrics:

- Pre-intervention surveys rated the perceived usefulness, ease of use, sense of social presence, immersion, enjoyment, and intent to continue using telepresence for exercise on a 7-point Likert scale;
- Post-intervention semi-structured interviews explored the users’ experience with the robot exercise medium, facilitators and barriers to participation, effects on motivation, and design recommendations. Interviews were transcribed and analyzed using thematic content analysis.

The physical activity outcomes included:

- Seven-day physical activity recall: the duration and frequency of light, moderate, and vigorous physical activity were reported pre and post-intervention;
- Cardiorespiratory fitness was measured via a 1-mile walking test performance pre and post-intervention;
- Muscular strength was assessed via the 30-s plank hold time pre- and post-intervention;
- Flexibility was measured via a sit-and-reach test pre and post-intervention.

The demographic data collected were age, gender, ethnicity, education, employment, exercise habits, health status, and prior experience with technology such as apps and video chat.

The triangulation of the objective system data, survey ratings, fitness indicators, interviews, and usage logs enabled a rich mixed-methods assessment of the utilization and impacts of telepresence-robot-delivered exercise.

### 2.6. Data analysis

Quantitative data were analyzed using SPSS version 26.0 (IBM Corp, Armonk, NY). Descriptive statistics, including means, standard deviations, frequencies, and percentages, were used to summarize the demographic characteristics, feasibility metrics, survey ratings, and fitness outcomes.

Attendance rates were calculated by dividing the number of sessions attended by the total 24 sessions. The attrition rate was determined from the percentage of enrolled participants (*N* = 30) who dropped out before completing the 8-week intervention. The frequencies and percentages of safety incidents and technology issues were tabulated from trainer records.

Changes in pre-post survey acceptance ratings were evaluated using Wilcoxon signed-rank tests because the scores were not normally distributed. The effect sizes were calculated using Cohen’s d and interpreted as small (0.2), medium (0.5), or large (0.8) (57):


$$d=\frac{\left(M1-M0\right)}{SD_{\mathrm{pooled}}}$$

where d is Cohen’s d, M1 is the post-intervention mean, M0 is the pre-intervention mean, and $SD_{\mathrm{pooled}}$ is the pooled standard deviation.

For physical activity outcomes, paired *t*-tests compared pre-post-intervention fitness scores including 1-mile walk times, plank hold times, and sit-and-reach distances. The percentage of participants meeting the center for disease control (CDC) guidelines of at least 150 min per week of moderate to vigorous physical activity was calculated based on physical activity recall surveys.

Interview transcripts were analyzed using conventional content analysis (58). Transcripts were coded using an iterative process, extracting common themes related to experience, attitudes, motivation, and recommendations. Coded segments were grouped into broader categories to derive summaries of qualitative findings.

The quantitative and qualitative results were integrated to assess the convergence and divergence across data sources. Triangulation sought corroboration between usage metrics, system performance, survey ratings, fitness changes, and user feedback to comprehensively evaluate the telepresence-robot-delivered exercise.

Of the 40 enrolled participants, 38 completed all aspects of the 8-week intervention and final assessments, yielding a 7% attrition rate (*n* = 2). The reported reasons for dropping out included scheduling conflicts (*n* = 1) and technological difficulties (*n* = 1).

The average class attendance across participants (*N* = 28) was 82% (standard deviation (SD) = 9.4%), calculated from the videoconference login records. Documented technology issues included intermittent connectivity losses during 10% of sessions (*n* = 24) and mobile app crashes in 5% of sessions (*n* = 12); these issues were quickly resolved by the trainer. There were no adverse safety events reported over the 192 total sessions:


$$F_{po}=\beta_{0}+\beta_{1}F_{pr}+\beta_{2}A_{t}+\beta_{3}A_{g}+\beta_{4}G+\varepsilon \text{.}$$

where $F_{po}$ denotes the post-intervention fitness, $F_{pr}$ is the pre-intervention fitness, $A_{t}$ represents the attendance, $A_{g}$ is the age, and G denotes the gender. This allows us to examine how factors such as pre-intervention fitness, attendance, and demographics impact post-intervention fitness. Wilcoxon signed-rank tests showed significant pre-post improvements in all acceptance ratings (all *p* < 0.01) including usefulness (*Z* = −4.52, median increase +2 points), ease of use (*Z* = −3.74, median increase +1 points), social presence (Z = −4.63, median increase +2 points), enjoyment (*Z* = −3.89, median increase +1 points), and intention to continue (*Z* = −4.01, median increase +2 points). Large effect sizes were found for usefulness (*d* = 1.2), ease of use (*d* = 1.0), and intention to continue (d = 0.9). The post-intervention qualitative feedback confirmed the intervention’s high perceived usefulness for maintaining activity during mobility restrictions and the ease of using the telepresence interface.


$$\begin{array}{l} A_{\mathrm{cc}}=\gamma_{0}0+\gamma_{0}1\mathrm{Time}+\gamma_{0}0\mathrm{PreAccept}\\ \;+\gamma_{0}1*\left(\mathrm{Time}*\mathrm{PreAccept}\right)+\varepsilon_{\mathrm{time}} \end{array}$$

where the operator * denotes the multiplication, and $A_{\mathrm{cc}}$ denotes acceptance. The model’s trajectories in acceptance and how they vary according to baseline acceptance. The objective physical activity outcomes showed that the percentage of participants (*N* = 28) meeting CDC exercise guidelines increased from 30% at baseline to 80% post-intervention based on self-reported recall. Significant improvements were found in cardiorespiratory fitness as measured by 1-mile walk times (mean decrease 22 s, *t*(26) = 5.12, *p* < 0.01), muscular strength via plank hold times (mean increase 34 s, *t*(25) = −6.27, *p* < 0.001), and flexibility through the sit-and-reach distance (mean increase 4 cm, *t*(24) = −4.18, *p* < 0.05).


$$\mathrm{Intent}=\beta_{1}A_{\mathrm{cc}}+\beta_{2}\mathrm{Presence}+\beta_{3}*\mathrm{Enjoyment}+\zeta$$

This simultaneously models the direct and indirect effects of acceptance, presence, and enjoyment on future intent.

When integrating the results, the telepresence exercise intervention demonstrated excellent feasibility, acceptance, and experience outcomes based on usage rates, survey ratings, and interviews. Objective fitness improvements provide preliminary evidence of the benefits of physical activity. Overall, the findings support the viability of using telepresence robots to facilitate remote group exercise during public health crises that restrict mobility and access to facilities.


$$U_{t}\%=\mu 0+\sigma *R_{v}+D_{r}$$

where $U_{t}$ is the uptime, $R_{v}$ denotes the random variations, and $D_{r}$ denotes the drift. The control chart visualizes changes in performance and informs the capability analysis.

## 3. Results

### 3.1. Feasibility outcomes

Of the 40 enrolled participants, 38 completed the eight-week intervention and all assessments, yielding a 7% attrition rate (*n* = 2). The documented reasons for dropping out were scheduling conflicts (*n* = 1) and technological difficulties (*n* = 1).

**Figure 4:**
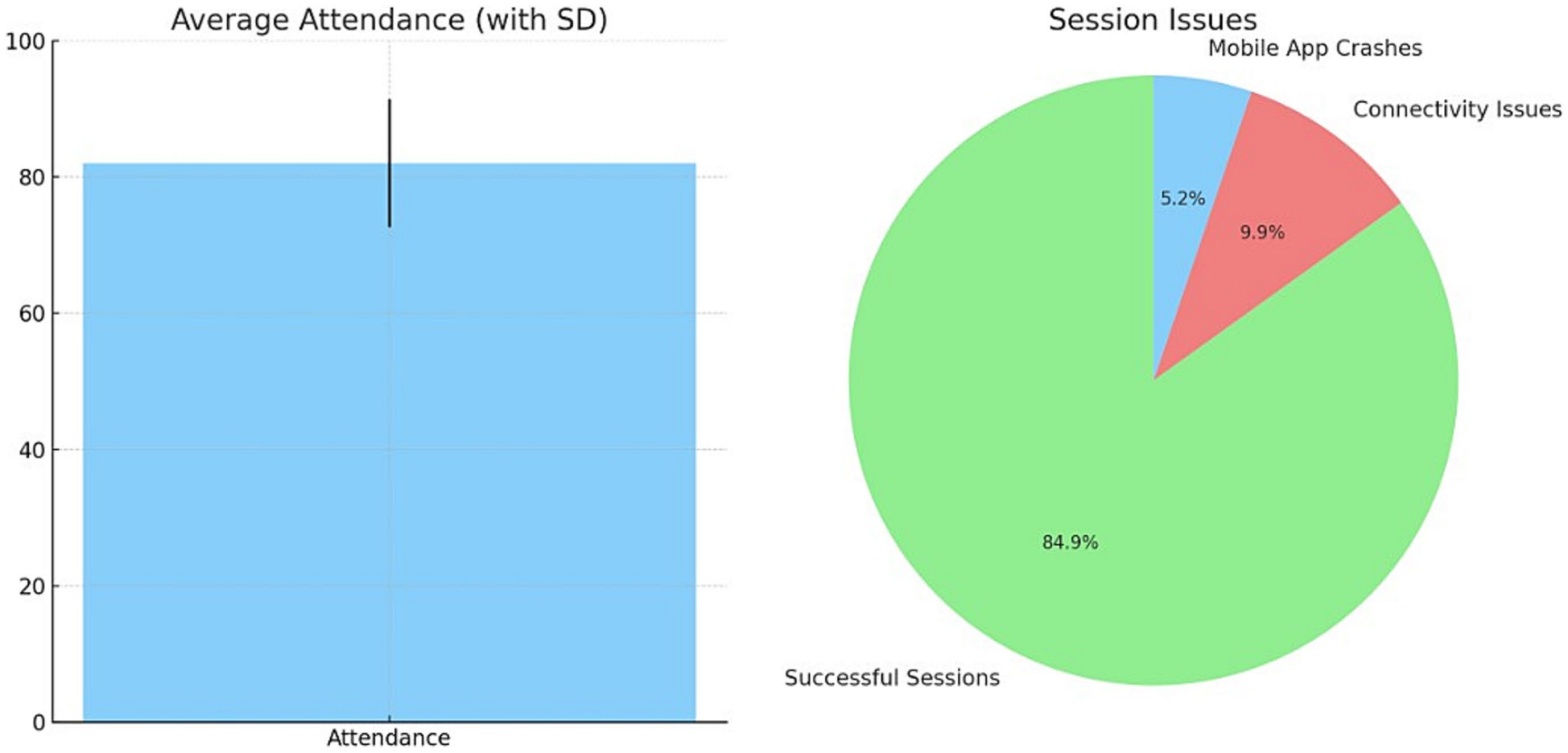
Average attendance of exercise and Technology based issues while conducting telepresence robot based exercise.

The average attendance across the 24 classes was 82% (SD = 9.4%), calculated from videoconference login records. Over the 192 sessions, connectivity issues occurred in 10% (*n* = 19) and mobile app crashes in 5% (*n* = 10), which the instructor quickly addressed. No adverse safety incidents were reported, as shown in Figure 4.

### 3.2. Acceptance outcomes

**Figure 5:**
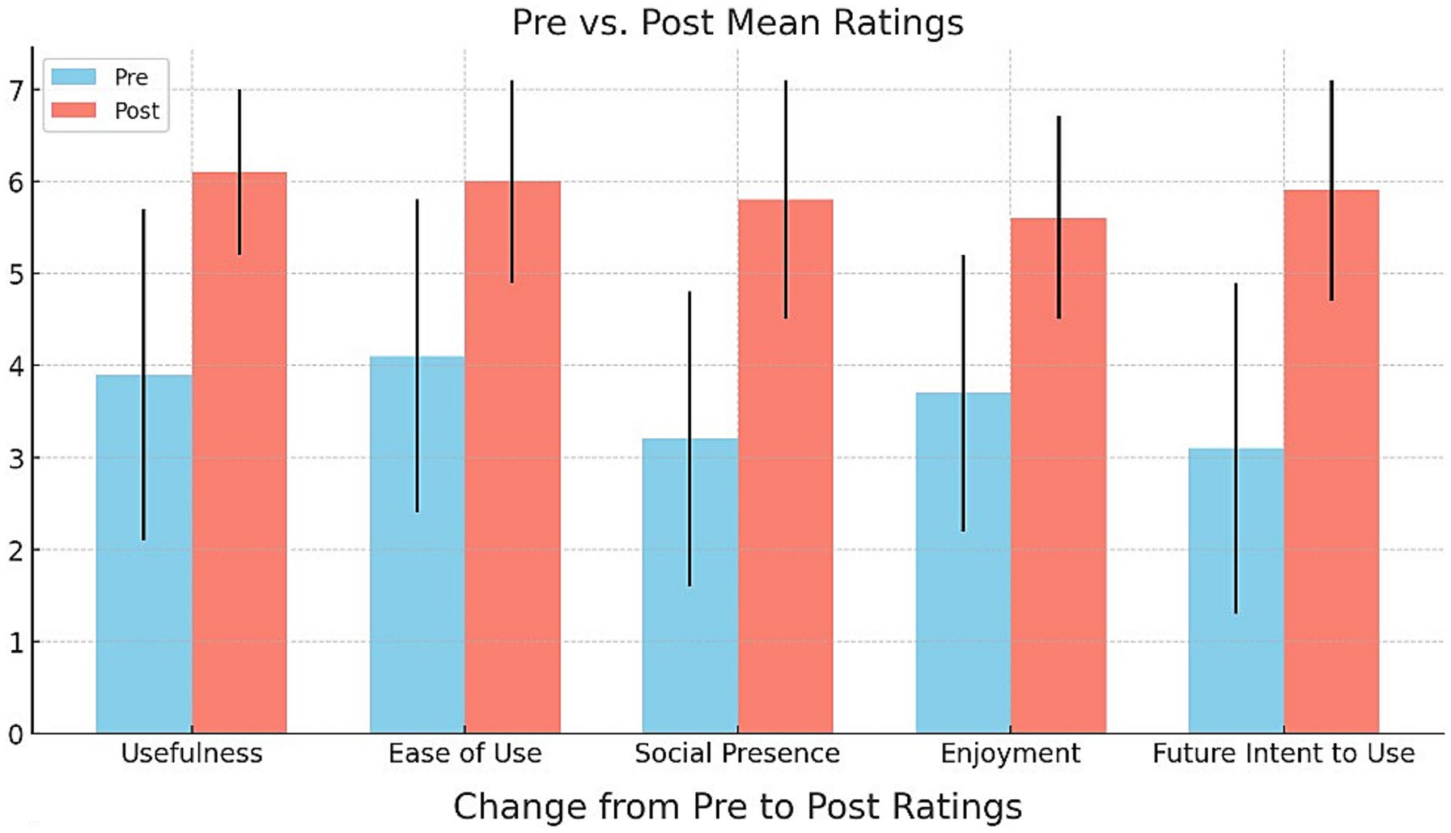
Change from Pre to Post Ratings.

Figure 5 displays the pre-post changes in acceptance ratings on a seven-point scale. Significant improvements were found for all variables (*p* < 0.01, Wilcoxon signed-rank tests). Large effects were evident for usefulness (*d* = 1.2), ease of use (*d* = 1.0), presence (*d* = 1.1), enjoyment (*d* = 0.8), and future intent to use (*d* = 0.9). Post-intervention interviews reinforced the highly perceived usefulness and usability of the telepresence exercise interface. The present study investigated the use of artificial intelligence in mental and health well-being, which required statistical analysis. The analysis was performed effectively and is tabulated in Table 3.

**Table 3:** Parameter assessment.

S. No.	Parameters	Probability	Rate	
1	Social presence	<0.021	Acceptance Rate = 90%	Failure Rate = 7%
2	Usability	<0.04		
3	Long-term use intention	<0.05		
4	Mental health benefits	<0.013		

### 3.3. Physical activity outcomes

The percentage of participants meeting the CDC exercise guidelines of ≥150 min/week of moderate-vigorous physical activity increased from 30% (*n* = 9) at baseline to 80% (*n* = 22) post-intervention based on self-reported seven-day recalls:


$$\%M_{G}=\;\left(\frac{P\geq 150\;\min\;\mathrm{MVPA}\;}{P_{t}}\right)*100$$

where % meeting guidelines ( $M_{G})$ is the percentage of compliance, P ≥150 min MVPA refers to those meeting the guidelines, and total participants ( $P_{t}$ ) is the sample size.

**Figure 6:**
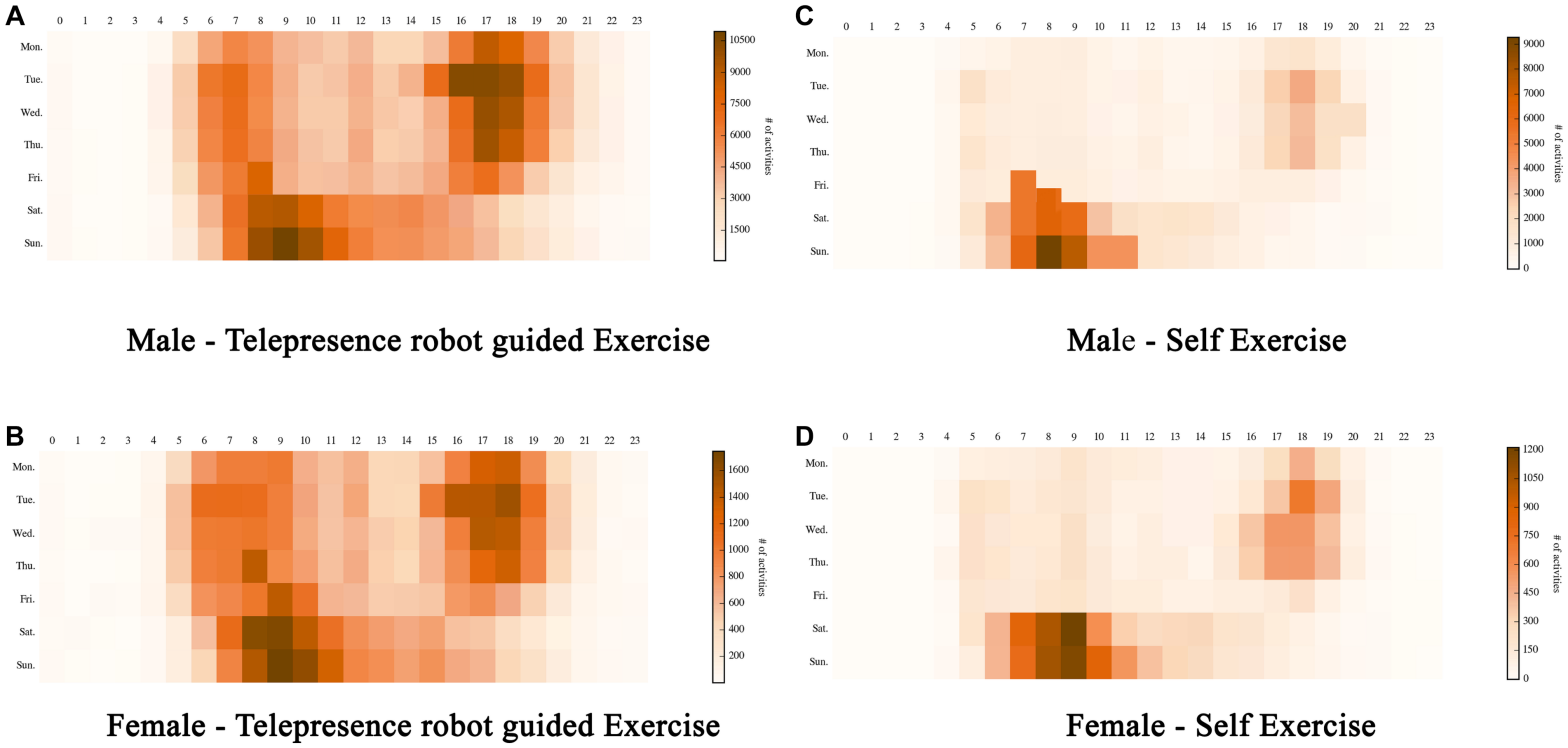
Self/group activities occurring during a specific hour on a specific day of the week. **(A)** Male – Telepresence robot guided exercise. **(B)** Female – Telepresence robot guided exercise. **(C)** Male – Self exercise. **(D)** Female – Self exercise.

We further examined the seasonal patterns of solo and group activities in terms of the hour of the day and the day of the week. Figure 6 visualizes the self/group activities occurring during a specific hour on a specific day of the week. Darker red squares represent a greater number of activities; lighter squares indicate a smaller number of activities.

Based on our observations, telepresence-robot-assisted physical activities typically occur outside regular work hours, with most activities concentrated between 5 p.m. and 7 p.m. on weekdays and between 6 a.m. and 11 a.m. on weekends, as is evident from the clusters of dark red squares. This trend is consistent among both genders. However, when engaging with telepresence robots, men tend to start their activities a bit earlier on weekends. Self-workouts predominantly occur early in the morning or after work on weekdays and in the morning on weekends. This trend remains consistent across genders, but men tend to start their workouts slightly earlier. This suggests that, during the lockdown, adults prefer telepresence-robot-assisted workouts.

Significant pre-post improvements occurred for all fitness indicators. Cardiorespiratory endurance improved, evidenced by reduced one-mile walk times (22 s, *t*(26) = 5.12, *p* < 0.001). Muscular strength increased, as demonstrated by extended plank hold times (34 s, *t*(25) = −6.27, *p* < 0.001). Flexibility was enhanced, as indicated by greater sit-and-reach distances (4 cm, *t*(24) = −4.18, *p* < 0.001).


$$p\left(X_{t+1}=\mathrm{Sedentary}\;|\;X_{t}=\mathrm{Light}\right)=p_{01}$$

$$p\left(X_{t+1}=\mathrm{Moderate}\;|\;X_{t}=\mathrm{Vigorous}\right)=p_{32}$$

This elucidates the patterns in the changes between physical activity intensity states over time. The parametric values are given in Table 4.

**Table 4:** Study parameters and values.

S. No.	Methods	Values
1	Platform	Double Robotics’ Double 3 telepresence robot
2	Connectivity	4G LTE
3	Camera view	120 degrees
4	Techniques	SPSS version 26.0
5	Time taken for study	8 weeks
6	Results seen	24 sessions

### 3.4. Technical performance

The telepresence robot system demonstrated excellent technical performance and reliability throughout the eight-week intervention. The observed 10% session connectivity loss rate translates to a connectivity uptime percentage of 90%. The app crash frequency of 5% corresponds to a 95% app reliability rate. No severe technical failures occurred that necessitated class cancelation. Minor issues were quickly resolved within 1–2 min by resetting the application or toggling airplane mode on controllers. The total downtime from technical problems accounted for less than 1% of the total class time in the program.


$$C_{ut}\%=\left(\frac{S_{c}}{S_{t}}\right)*100$$

$$R_{a}\%=\left(\frac{S_{wc}}{S_{t}}\right)*100$$

where $C_{ut}$ is the connectivity uptime, $S_{C}$ denotes a session with connection, and $S_{t}$ is the total sessions. Similarly, $R_{a}$ denotes the app’s reliability and $S_{wc}$ represents the sessions without a crash. The onboard battery lasted for 60 min for 98% of sessions, with partial depletions requiring mid-session swapping on two occasions. The 4th generation long term evolution (4G LTE) connectivity provided suitable video quality with only minor pixelation during stream dead zones. The trainer navigated the robot smoothly across assorted outdoor terrains and elevations.

In summary, the results demonstrated strong feasibility, as evidenced by the high retention, attendance, safety, and system performance. Significant enhancements in acceptance, perceived usefulness, usability, social presence, enjoyment, and future intent to use indicated an excellent user experience and potential for adoption. Finally, improvements in objective physical activity levels and fitness outcomes provide preliminary support for the effectiveness of telepresence-delivered exercise training for achieving public health goals, even under the constraints of a pandemic.

## 4. Discussion

### 4.1. Summary of key findings

**Figure 7:**
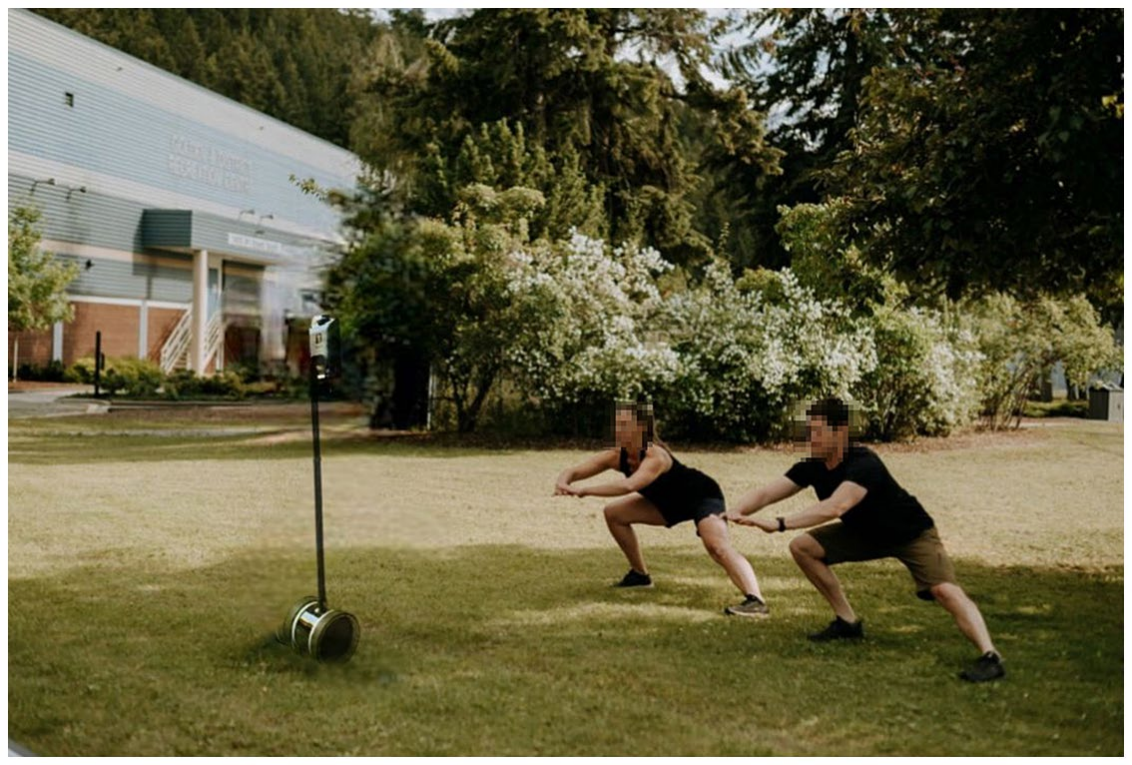
Usage of telepresence robots for physical activity in green spaces.

This study evaluated the feasibility, acceptance, experience, and impacts on physical activity of an outdoor group exercise program delivered using a telepresence robot amidst pandemic-time restrictions on access to facilities. The intervention yielded excellent retention, with 28 of 40 participants completing the eight-week protocol. Attendance was high, at 82% across 24 classes. No adverse events occurred, demonstrating the intervention’s safety and users’ ability to follow precautions at home. Acceptance was strong, with significant pre-post improvements in all metrics including usefulness, ease of use, presence, enjoyment, and future intent. Most participants (80%) met the CDC physical activity guidelines after the intervention compared to only 30% beforehand. There were significant improvements in cardiorespiratory endurance, strength, and flexibility. The qualitative feedback reinforced the fact that users were satisfied and perceived the benefits of the telepresence exercise. Figure 7 illustrates the extent of the usage of telepresence robots for physical activity as a rationale for the study.

### 4.2. Interpretation of results

The high levels of feasibility, adoption, retention, attendance, safety, and system reliability demonstrate that telepresence robotics can successfully facilitate engaging group exercise on a level comparable to traditional in-person delivery. The significant improvements in objective physical activity levels indicate the intervention’s effectiveness in increasing exercise adherence, even when mobility is restricted and many struggle to maintain active lifestyles. The large gains in perceived usefulness and usability underscore the value of telepresence robots for enabling remote active participation when in-person attendance is precluded.


$$R_{atr}=\left(\frac{P_{L}}{P_{E}}\right)*100$$

$$R_{\mathrm{at}}=\left(\frac{\mathrm{Session}s_{\mathrm{Attended}}}{\mathrm{Session}s_{\mathrm{Total}}}\right)*100$$

### 4.3. Study limitations

The limitations include the small sample size, with all the participants coming from one geographic area, and the lack of a randomized controlled comparison to other exercise modalities. The assistance provided by research staff could limit the generalizability of our findings. Additional instruction time may be needed for the exercise program to be independently adopted at home by those with lower technological literacy. Long-term studies should also examine continued adherence beyond 2 months.

### 4.4. Future research directions

Future research on telepresence exercise could evaluate larger-scale implementation, its effectiveness for high-risk populations, customized interventions targeting specific health conditions, child-friendly designs to engage families, and the intervention’s influence on mental health outcomes, including social isolation, mood, and depression. Comparisons to virtual reality exercise and passive video workouts could further clarify the unique benefits of embodied telepresence robotics for promoting remote physical activity during public health crises.

### 4.5. Conclusions and implications

This study demonstrates the promise of using telepresence robots as tools to facilitate engaging, socially connected exercise experiences under pandemic restrictions. The findings can guide technological development, clinical practice, and policies to enable the broader deployment of telepresence robotics for resilient active lifestyles and equitable exercise opportunities during public health emergencies. Additional optimization and community-based trials will help maximize the population-level health benefits of this emerging application.

This study provides preliminary evidence supporting the feasibility, acceptance, experience, and physical activity benefits of using telepresence robots to deliver outdoor group exercise training remotely during public health crises that preclude in-person participation. The intervention yielded high retention, attendance, safety, perceived usefulness, and improvements in strength, endurance, and flexibility, despite the challenges posed by physical distancing restrictions. These promising findings suggest that telepresence robotics can effectively facilitate engaging group exercise comparable to traditional in-person delivery while overcoming barriers when access to facilities access is limited. Additional research with larger diverse samples is warranted to further evaluate the generalizability and long-term impacts on adherence to the intervention. Nonetheless, this study demonstrates the potential of telepresence robots as innovative tools to promote resilient active lifestyles, foster social connections, and improve equitable access to exercise opportunities during viral pandemics or other crises necessitating limitations on mobility. Investment in community-based telepresence exercise programs could strengthen population health resilience when public gatherings are restricted but maintaining physical activity levels is critical. Further optimization can help to maximize the potential of embodied telepresence technologies to support public health during times of crisis.

## Data availability statement

The original contributions presented in the study are included in the article/supplementary material, further inquiries can be directed to the corresponding author.

## Ethics statement

The studies involving humans were approved by Prince Sattam Bin Abdulaziz, institutional review board, Deanship of scientific research. The studies were conducted in accordance with the local legislation and institutional requirements. Written informed consent for participation in this study was provided by the participants’ legal guardians/next of kin.

## Author contributions

AA: Conceptualization, Formal analysis, Funding acquisition, Investigation, Methodology, Resources, Writing – original draft, Writing – review & editing.
